# Practical guide for the use of medical evidence in scientific publication: Recommendations for the medical student: Narrative review

**DOI:** 10.1016/j.amsu.2021.102932

**Published:** 2021-10-09

**Authors:** Ivan David Lozada-Martínez, Laura Marcela Acevedo-Aguilar, Laura Marcela Mass-Hernández, Duván Matta-Rodríguez, Jhoyner Alberto Jiménez-Filigrana, Karen Elizabeth Garzón-Gutiérrez, Sergio Antonio Barahona-Botache, Danna Lianeth Vásquez-Castañeda, Sharon del Rosario Caicedo-Giraldo, Sabrina Rahman

**Affiliations:** a Medical and Surgical Research Center, School of Medicine, University of Cartagena, Cartagena, Colombia; b School of Medicine, Universidad de Ciencias Aplicadas y Ambientales, Bogotá, Colombia; c School of Medicine, Universidad El Bosque, Bogotá, Colombia; d School of Medicine, Fundación Universitaria Juan N. Corpas, Bogotá, Colombia; e School of Medicine, Universidad Surcolombiana, Neiva, Colombia; f School of Medicine, Universidad Tecnológica de Pereira, Pereira, Colombia; g School of Medicine, Universidad del Tolima, Ibagué, Colombia; h Independent University, Dhaka, Bangladesh

**Keywords:** Evidence-based medicine, Research, Bias, Medical student, Scientific publication

## Abstract

Currently, the adaptation of scientific evidence in clinical problem solving is based on the evidence-based medicine method. Medical students and health professionals should have an adequate knowledge of this method and thus provide adequate health care and increasingly provide high quality scientific publications that can be subsequently integrated in different clinical scenarios. Several scales and tools have been proposed as guides to recognize the different levels of quality of the available evidence, their degrees of recommendation and the biases and fallacies that may occur both in the clinical and research areas, with the aim of identifying the best available evidence. However, few students and professionals are aware of them and make proper use of them. Therefore, it is necessary to synthesize these tools in an understandable and practical way.

## Highlights

• The student's knowledge and use of medical evidence is indispensable.
• It is necessary to train the medical student in scientific publication.
• The medical student must recognize biases and levels of evidence.

## 1 Introduction

Evidence-based medicine represents the method for integrating scientific evidence across the various fields of biomedical sciences to support rational clinical practice, responsible decision making, and appropriate use of diagnostic and therapeutic tools, based on the results of systematic studies conducted under a rigorous methodology [[1], [2], [3], [4], [5]]. This approach constitutes an important advance in the area of medicine, since being a science, it requires reliable knowledge to guide an adequate development of medical practice and with this, provide better patient care and obtain satisfactory outcomes in the short- and long-term [6]. As a constantly evolving science, there is an active dynamic where evidence from different levels emerges rapidly, which makes it necessary to continuously synthesize such results in order to verify which interventions are the most favorable and cost-effective in practice. For this reason, it is essential for every physician to recognize and make appropriate use of scientific evidence [7]. (see Table 3)

The medical student is an actor that in the last decade has gained great relevance in this process, since it participates more and more actively in the creation of evidence through scientific research from medical schools, contributes to the design of strategies for the improvement of medical education, cooperates with the dissemination of scientific knowledge in events of social appropriation and circulation of new knowledge, and has become more critical regarding the outcomes of global health [[8], [9], [10], [11], [12], [13], [14]]. This has led students to be interested in deepening their knowledge of scientific evidence in order to acquire knowledge and tools that allow them to propose solutions to public health problems [10,12,13]. However, evidence in medicine is a broad and complex field, which requires a great deal of experience and academic preparation to be able to identify it precisely. Nevertheless, this does not mean that the student does not need basic or advanced notions about research, critical reading of scientific texts, quality of evidence and scientific publication, among other things [2,8].

Although there are studies that have demonstrated the substantial and positive impact of medical research and scientific publication during undergraduate medical school on the professional career [[15], [16], [17], [18], [19], [20]], there is also evidence that supports the fact that most of these students do not understand the evidence, see it as an impossible topic and prefer to avoid it; a problem that must be solved [[21], [22], [23], [24]]. The step-by-step participation in scientific publication, from the lowest levels of evidence, is an intelligent strategy that allows the student to consolidate knowledge about critical reading, use of evidence and the editorial process of medical journals, which translates into an understanding of the dynamics of evidence in medicine [[11], [12], [13]]. However, it is necessary that the student possesses basic concepts that facilitate the understanding of the evidence and allow him/her to satisfactorily interpret the results of scientific texts.

Based on the above, the objective of this review is to design a practical guide for the use of evidence in scientific publication for medical students, to serve as a basis for their training and practice in research and scientific publication in medicine.

## 2 Methods

A non-systematic literature search was carried out in the PubMed and Scopus databases, using the keywords “medical evidence”, “scientific publication”, “medical student”, “evidence-based medicine”, and synonyms, which were combined with the Boolean operators AND and OR. The search was performed until July 2021. Clinical trials, original studies, reviews, commentaries and letters to the editor were included. The only exclusion criterion was the unavailability of the full text. Finally, 48 articles were included. Additionally, a reverse search was performed, finding 16 articles, finally using 64 references.

### 2.1 Basic considerations and advances in evidence in medicine

The term “evidence-based medicine” emerged in the early 1990s through a campaign proposed by a movement formed by a group of epidemiologists from McMaster University in Canada, known as the “evidence-based medicine movement” [25,26]. The main objective of this movement was to develop a novel model to achieve the highest degree of objectivity in clinical problem-solving decisions in practice [26]. Thus, this group of epidemiologists led by Dr. David Lawrence Sackett, published at that time several guidelines on the role of evidence-based medicine in medical education [27], with emphasis on learning about study designs so that students and professionals could make critical evaluations of published studies, their results, the veracity of the data and the degree of recommendation of that evidence [7]. Subsequently, the “Cochrane Collaboration”, founded in the United Kingdom, was formally established with the aim of providing and gathering the best evidence on health issues, constituting the most outstanding result of the evidence-based medicine movement to date [8].

This process allowed the birth of scales to assess study biases and determine how valid the results were within the study design (known as internal validity), and how feasible it is to extrapolate those results to the general population (known as external validity) [[26], [27], [28], [29]]. It allowed the birth of scales to categorize the level and degree of recommendation of the evidence; that is, how reliable are the results based on the study design and how likely it is that these results are applicable in the practical context [30,31]. However, during the analysis of the evidence, other factors were also found to influence the veracity of the results and their respective interpretation, namely the presence of fallacies, which are very common among the general community, but have a negative impact on the responsible use of evidence [28]. In this order of ideas, one of the objectives of medical education is also to find ways to integrate the learning of evidence-based medicine from the undergraduate level, to achieve satisfactory results in professional practice, and to create academic and research profiles for students. Over the past few years, the results of the progress of evidence-based medicine have been demonstrated through increasingly comprehensive and detailed national and international databases, improvements in final and functional outcomes of disease management, availability of better quality evidence, and incorporation of medical students and general practitioners in international collaborations and international scientific societies [[28], [29], [30]]. Likewise, through publications such as commentaries and letters to the editor, the authors' interest in constructive criticism of public evidence is reflected.

However, in order to adopt this approach in the context of medical practice, a series of steps must always be followed: The first is to establish a clinical question; subsequently the best existing evidence about it must be sought; then it is required to assess the results according to their level of evidence; apply them in practice according to the clinical judgment of the health professional, and finally re-evaluate the performance of the entire process [32]. Steps in which constant accompaniment and training are needed by the medical student and medical professional. Participation in research projects and scientific publication are basic recommendations of evidence-based medicine centers to improve the quality of the professional and of the evidence [12]. But also, the knowledge of tools for the validation of evidence is a must.

### 2.2 Evidence grading scales, uses and interpretation

It can be said that the scales of evidence-based medicine are the first tool that the student should know. This will allow them to obtain updated and quality information from the study of their academic content in basic sciences, to the analysis of decision-making algorithms and therapeutic decisions during their clinical practice. This is because not all studies have the same weight to support a good recommendation in the clinic [31]. Currently, there are multiple classification systems to evaluate these factors, among which the Oxford Centre for Evidence-Based Medicine (CEBM) system [33] and the GRADE (The Grading of Recommendations Assessment Development and Evaluation) system [34] stand out [34].

#### 2.2.1 Oxford university evidence-based medicine scale

This classification system is based on evaluating the evidence according to the different clinical scenarios and types of studies [35]. Because of this, it provides a more specific knowledge of each scenario, which is a great benefit when integrating with clinical decision making [31]. However, it also has certain disadvantages since it uses complex methodological and epidemiological terms that may be difficult to understand for readers who are not completely familiar with them [35].

The hierarchy of the levels of evidence consists of a list from 1 to 5, where there are sub-levels **(a, b or c)**, with level 1 corresponding to the best available evidence, and level 5 to the lowest quality [33,35] (Table 1). In total, there are 10 levels of evidence according to this scale (see Table 2).

**Table 1:** Summary of CEBM system levels of evidence for diagnostic, prognostic and interventional outcomes [33].

Level of evidence	Diagnosis	Prognosis	Therapy-prevention-etiology-damage
1a	Systematic reviews of high quality diagnostic studies with homogeneity; studies with comparable products from different clinical centers	Systematic reviews of initial cohort studies with homogeneity; studies with comparable approved products in different populations	Systematic reviews with homogeneity of randomized clinical trials
1b	Quality-validated cohort studies with good reference standards	Individual cohort studies with a follow-up greater than 80%; validated in only one population	Single clinical trial with narrow confidence interval
1c	Diagnostic tests with high specificity and sensitivity	Case series	All or none
2a	Systematic reviews with homogeneity of level 2 diagnostic studies	Systematic reviews with homogeneity of retrospective cohort studies or control groups not treated with clinical trials	Systematic reviews with homogeneity of cohort studies
2b	Exploratory cohort studies with good baseline standards	Retrospective cohort studies or follow-up of untreated controls with clinical trials or unvalidated practice guidelines	Individual cohort studies with less than 80% follow-up; including low quality clinical trials
2c	None	Health outcomes research	Ecological studies; health outcomes research
3a	Systematic reviews of studies 3b and better quality with homogeneity	None	Systematic reviews with homogeneity of case-control studies
3b	Non-consecutive studies or studies that do not have the application of reference standards	None	Individual case-control studies
4	Case-control studies with few or no non-independent benchmarks	Case series; cohort studies of low quality	Case series; case-control and cohort studies with poor quality
5	Expert opinion that has no explicit critical evaluation, nor is it based on physiology, research or elementary principles.	Expert opinion that has no explicit critical evaluation, nor is it based on physiology, research or elementary principles.	Expert opinion that has no explicit critical evaluation, nor is it based on physiology, research or elementary principles.

**Table 2:** Grade of recommendation system CEBM [33].

Grade of recommendation	Interpretation
A	Studies belonging to evidence level 1
B	Studies belonging to level of evidence 2 or 3
C	Studies belonging to evidence level 4
D	Studies belonging to evidence level 5 or inconclusive or inconsistent studies of any level

**Table 3:** GRADE system evidence quality levels [34,36].

Quality level of evidence	Study design	Interpretation
High	Randomized clinical trials, systematic reviews and meta-analyses	High level of confidence in the effect of the intervention
Moderate	Observational studies	There is confidence in the effect of the intervention, however, there may be future studies that modify the effect
Low		Confidence in the effect of the intervention is likely to change with future studies
Very low	Any other type of evidence (case reports, case series, commentaries)	There is no certainty about the effect of the intervention

### 2.3 Sistema GRADE

The GRADE system is a classification that proposes a systematic approach based on the definition of various criteria in order to make judgments about the quality of the evidence and the strength of its recommendation [[34], [35], [36]]. The level of quality of evidence is classified into 4 categories: high, which includes experimental studies; moderate and low, which includes observational studies; and very low [30].

On the other hand, with respect to the grades of recommendation, this system takes into account the balance between benefits and risks, the quality of the evidence, its application in particular clinical scenarios, taking into account the values and preferences of each patient, and the use of resources [37].

These scales behave as a guide, since there are aspects such as biases and fallacies, which compromise the level of evidence of the study design and alter the degree of recommendation. Therefore, the scales are not an absolute (see Table 4).

### 2.4 Correct use of evidence in medicine and scientific publication: the importance of knowing the biases

Medical errors in clinical practice decision-making lead to a large number of unnecessary hospitalizations, excessive use of drugs and resources, increased healthcare costs and do not contribute to the improvement of the overall burden of disease [38]. Such errors in clinical reasoning are often attributed to cognitive biases [39], which are defined as any tendency that hinders the unbiased consideration of an inquiry [40]. Biases compromise physician judgment, and therefore, timely recognition by medical personnel of potential biases in evidence and practice is extremely important in order to make medical decisions with favorable and meaningful outcomes, thus reducing malpractice errors [41]. However, these biases should primarily be identified by the author and the reader of the scientific publication or the user of the evidence, in order to prevent others from making mistakes. The literature describes innumerable biases in medicine, some of which share applicability both in practice and when creating or interpreting evidence (Table 5). Although it is common to observe these biases among the general population, their permanence in medicine can lead to catastrophic results, whether at the professional, institutional or community level.

**Table 4:** Grades of recommendation of the GRADE system [34,36,37].

Grade of recommendation	Interpretation
1A	Strong recommendation; high quality level
1B	Strong recommendation; moderate quality level
1C	Strong recommendation; low or very low quality level
2A	Weak recommendation; high quality level
2B	Weak recommendation; moderate quality level
2C	Weak recommendation; low or very low quality level

**Table 5:** Biases that can occur in medical practice [39,40].

Bias	Definition	Example
Anchoring	Anchoring to a diagnosis	Perform antifungal therapy to a ring-like lesion, and it turns out to be a case of discoid lupus erythematosus
Availability bias	Favor readily available solutions	Make diagnoses based on previous patients with similar clinical pictures
Framing effect	To favor a decision according to the information presented	Assume that the symptoms of a patient coming from Africa correspond to the clinical picture of malaria
Premature closure	Do not continue to seek additional information after establishing a diagnostic conclusion that has not been fully verified	Treating pneumonia in a patient with acute dyspnea, not investigating further and consequently not recognizing secondary myocardial infarction
Confirmation bias	Tendency to prefer certain findings in the information provided, in order to confirm a diagnosis	Suspect that the patient has an infection and the elevated leukocytes confirm this, rather than questioning why the leukocytosis is occurring
Diagnostic momentum	Continue actions instigated by previous clinicians without taking into consideration available information and make adjustments if necessary	Continuing a clinical course for pulmonary embolism in a patient with subsequent findings suggesting no such pathology

In addition, the existence of implicit biases has been established, which occur between the attributes of a particular group and a negative evaluation of that attribute by health professionals without being aware of it, which can then perpetuate inequalities in the health system, having an unfavorable impact on patients from stigmatized groups (e.g., people with low socioeconomic resources, minority ethnic groups, the disabled, among others) [[42], [43], [44]]. Education in evidence-based medicine develops the physician's prudence and reasonableness to reduce the risk of these biases, since it recognizes that evidence must be handled and expressed with care and great detail.

On the other hand, in the area of scientific research and publication, biases can also appear during any of the stages of the research, being able to cause distortions in the estimations of associations and correlations (Table 6) [40]. Although these biases are many and some do not depend on the author of the publication or the reader, implicitly it should always be kept in mind that the evidence is supported by statistical models and manifests itself in probabilities, therefore, nothing is absolute. Nevertheless, one should try to look for and obtain the strongest and best quality evidence.

**Table 6:** Summary of some biases in scientific research [40].

Bias	Definition
Selection bias	When the selection criteria used in separate cohorts of studies are inherently different
Interviewer bias	Systematic discrepancy in the way information is solicited, recorded and interpreted
Chronological bias	When controls from long past times are used as a comparison group with patients exposed to clinical behavior
Citation bias	When researchers do not publish unfavorable results in their studies, in order to avoid having the efficacy of the study negatively questioned

#### 2.4.1 Scales and tools to recognize biases

The presence of biases significantly reduces the certainty and significance of the results obtained by the studies and given to the recommendations, therefore, they do not guarantee the effect of an intervention and must be recognized in order to avoid errors in clinical practice. In this order of ideas, it is important to reinforce knowledge about biases in medical students and health professionals in order to improve medical care and to critically review the available scientific evidence [38]. To this end, tools have been developed that have shown successful results in the context of recognizing different biases [41]. These tools vary with respect to the type of bias and must be adjusted to the particular characteristics of each type of study (Table 7) [45,46]. These tools are necessary when synthesizing evidence, such as in systematic reviews and meta-analyses, where acceptable homogeneity must be guaranteed. Therefore, during the reading of these studies, the bias score of the synthesized studies should be explicitly stated, thus estimating the reliability of the estimates.

**Table 7:** Example of some tools for recognizing biases [45,46].

Tool	Types of studies
Cochrane risk of bias tool for randomized trials (RoB 1.0) y RoB 2.0 revised tool for assessing risk of bias in randomized trials	Randomized controlled trial
Risk Of Bias In Non-randomized Studies of Interventions I (ROBINS-I) tool	Non-randomized intervention studies
Agency for Healthcare Research and Quality (AHRQ) outcome and analysis reporting bias framework	Cross-sectional studies/prevalence
Quality In Prognosis Studies (QUIPS) tool	Prediction studies
Appraisal tool for Cross-Sectional Studies (AXIS) tool	Cross-sectional studies
EPOC RoB tool	Randomized controlled trial; controlled clinical trials; interrupted time-series studies; controlled before-and-after study
Systematic Review Centre for Laboratory animal Experimentation (SYRCLE) RoB tool	Studies with animals

### 2.5 Use of fallacies in medicine and scientific publication: the deadly enemy of evidence

It is difficult to define the term “fallacy” in a unified definition, since over time many concepts have been attributed to it. However, it basically refers to an argument that seeks to persuade or defend what is false; in other words, it is a lie, a deception or an invalid reasoning [47]. Fallacies are present in our daily lives more than we think and we can use them very often without realizing the power they have to make illogical arguments seem logical [48]. In the health field, especially in emergency situations, physicians are under pressure to act promptly, so they may feel obliged not to make mistakes, to want to know everything and to hide their ignorance, which encourages them to use fallacies; this, added to the fact that many scientific publications are based on fallacies that place the lives of thousands of patients at potential risk, due to conflicts of interest [49]. These should also be studied and known by both the medical student and the health professional (Table 8), in order to try to obtain objective evidence with less risk of bias in their results.

**Table 8:** Types of fallacies most commonly used in the clinical and research area [50].

Fallacy	Definition	Examples
Post hoc ergo propter hoc	We assume that a first event is the cause of a second event, simply because it happened before the second event	“I was sick, I received treatment and now I am cured. The treatment was the cause of my recovery."
Argumentum ad verecundiam (also known as eminence-based medicine)	Argument proposed as true because the source of the information is considered an expert or authority	- “It must be true because the surgeon said so." - “This treatment is completely effective because The Lancet published a study recently."
Argumentum ad populum (“Everybody says so”)	Argue that a fact is true simply because many or most people believe it to be so	“Alternative medicine is useful because many people claim it and practice it"
Argumentum ad ignorantiam	Conclude that a fact is true on the basis that it is not known to be false, or vice versa	There is no evidence that therapy with ARA II or ACEI increases the risk of coronavirus disease. Thus, the assumption that such therapy increases risk should not be true
Simple explanation	Belief that every complex problem has a simple explanation	Argue that the cause of coronary heart disease, cancer, peptic ulcers, and many other disorders are caused by “stress"
The faggot fallacy	Belief that multiple elements considered weak or unsafe separately, when grouped together provide solid evidence	Evidence based on a series of studies in which no significant results were obtained
Extrapolation from one condition to another (False dilemma)	It is limited to two options or possibilities when in reality there are others	It is recommended that patients who are taking ACEI or ARB II for the control of high blood pressure, heart failure or other indications not withdraw their treatment regimens unless instructed to do so by their physician
The bad-blood fallacy	Certain pathologies are considered to be strictly related to blood groups	There is a close relationship between people with blood group O and duodenal ulcer
The ecological fallacy	Characteristics of a population are attributed to an individual	“Women with a high-fat diet are more likely to develop breast cancer, because studies show that breast cancer mortality rates are higher in countries where fat consumption is high."
The fallacy of “positive results” (Type 1 error)	Simply presenting the “positive” results of a study, when in reality there were no effective results in the populations studied	10 research groups conduct a study of a new treatment for schizophrenia. 6 groups found no demonstrable effect, 2 groups obtained toxic effects and 2 others showed some degree of benefit. These last two studies are published because they show “positive” results”.
The fallacy of experience	To set aside critical appraisal, sound experiments, evidence and resort to personal experience	An oncologist observes a good remission in an advanced cancer after using a certain chemotherapy treatment and now applies the same treatment to all of his cancer patients without thinking about the consequences.
The fallacy of obfuscation	Use of language to disorient or confuse, rather than allow for clear understanding	A physician who does not write clearly or who uses language that is too technical and confuses his patients
The “hush, hush” fallacy	Ignoring the fact that mistakes are inevitable	A surgeon who prefers to hide his mistake so as not to jeopardize his authority as a specialized health professional, putting his patient's life at risk
The fallacy of the gold mean	Assuming that the consensus of a group of experts indicates the truth	The use of ivermectin or anticoagulants by “expert” recommendation during the COVID-19 pandemic, in the absence of evidence

### 2.6 Scientific publication during undergraduate medical school: advantages and difficulties

Several studies have shown that those medical students who research and publish before graduation tend to continue publishing after graduation and even publish much more than those who begin their research involvement as physicians [16]. Because of this, the best way to introduce a culture of evidence-based medicine to future physicians is by encouraging scientific inquiry in undergraduate students [51]. This is a vital tool in the field of medicine, as it allows the student to obtain critical thinking skills, broaden their knowledge [19], grow in terms of personal satisfaction, recognition and academic experience, learn keys to enrich their curriculum [8], acquire advantages to enter competitive residencies [52], get a better job and professional stability.

Despite the enormous importance of scientific skills, the number of student researchers is very low, mainly due to the lack of scientific training and incentives in their faculties [53], added to multiple factors that limit scientific research, such as lack of student time, difficulty in choosing a topic, few tutors, among others [52]. Beyond methodological factors, there are different problems facing the world of scientific research, among which is the inequality of opportunities between men and women [54,55], with a greater number of articles published by men [15].

On the other hand, there is the “scientific racism” of scientific journals, where some of them undervalue the work done by the medical student, and in order to maintain a high impact factor and h-index, they prefer to accept manuscripts written by recognized professionals with high metric indicators, regardless of the research value of the other manuscripts received [56]. In addition, there is also the selectivity of articles according to the nationality of the authors, where scientific papers from low- and middle-income countries are undervalued, due to scarce funding, low prestige of the authors, low impact of national journals and language limitations [8].

### 2.7 Recommendations when writing a scientific article

Knowing the levels of evidence, biases, fallacies, quality of information and the relevance of training as a research-based physician, the basic scientific writing process also allows to recognize errors during the process of creating and publishing a scientific article. Some of the recommendations to take into account when carrying out a scientific publication is that during the literature search, references should be as recent as possible and obtained from databases such as PubMed, MEDLINE, Scopus, Web of Science (WoS), Science Citation Index (SCI), or Scielo (for Latin America and the Caribbean), which have metrics that guarantee less bias in the publication of articles in indexed journals [57]. The acronym IMRaD (Introduction, Methods, Results and Discussion) is the format usually used in the preparation of scientific articles, although each journal has certain instructions for authors, so it is possible to find small variations in the format of each of them, as well as the number of words and references, depending on the type of study to be carried out [58].

The title of the article should be neither too short nor too long, ideally a concise and complete title, easy to read and characterizing all the information provided in the article, to arouse the reader's interest [59]. The abstract should contain the most relevant information, almost always limited to 250–300 words organized in a precise manner, since it is often the only section of the article that is read [57]. The introduction should contextualize the reader, cover the relevance of the subject and objective of the study, and provide general background information on the subject [58]. The methods contain the elements that will guarantee the reproducibility and falsifiability of the study, to determine the quality of the results. Therefore, here it should be explained in detail how the study was conducted, how the selection of participants, data collection, sampling, dependent and independent variables, among other points, so that readers can analyze the results adequately [60].

On the other hand, in the results section, the key findings of the study are presented, one by one, in an objective manner, using tools such as graphs or tables [61]. Finally, the discussion provides a subjective interpretation of the main findings, comparing them with previously published evidence, and highlights the novel points of the manuscript [62]. Additionally, the discussion should include the limitations of the study, as well as the implications of the relevant findings in clinical practice [59]. The conclusion can go at the end of the discussion or it can be added as a subtitle, and in it a last message is presented, in a concise form, where the reader receives the message about the evidence found in the study [57]. In addition, recommendations, comments or reflections of the study can be added for future scientific research [60].

It is necessary to always keep in mind that the process of scientific writing is a road full of obstacles, where many times you will fall and will not be able to express your ideas in the best way. However, the more you travel this road and learn to use technical language, to interpret evidence, to collect and understand estimates, among other items, the easier the road will be to overcome. The medical student may be discouraged at the beginning of this journey, but should remember that at the end of the road, there is a priceless treasure, the knowledge and expertise to use evidence to their advantage, and it is the key that can guarantee good final results as a professional.

### 2.8 Current goals and future challenges of evidence and global medicine

One of the main goals of evidence-based medicine is the stimulation or early motivation of medical students in the field of research by the different academic entities, based on a formal scientific education, where students participate actively, in order to achieve a real commitment on the part of these students [16]. There are Cochrane collaborations for medical students or student chapters in scientific societies, which help train future physicians in the effective use of evidence-based medicine [63].

A fundamental strategy to achieve this objective is the collective establishment of interest groups [10,[12], [13], [14]], active academic communities that through workshops, seminars, tutorials, research projects and other activities, allow the development of multiple skills in students, through scientific publication, participation in scientific events, presenting research results and proposing practical solutions to local or national public health problems [64].

In this order of ideas, one of the main objectives would be the design of a mixed curriculum that includes a strong component of scientific research for medical students, which facilitates their approach to scientific evidence. There is a need for universities to liaise with student societies and to encourage students who stand out for their commitment to evidence-based medicine, and to recognize the value of evidence-based medicine in the academic community. Financial support, infrastructure, academic content and teaching support during the design and maintenance of interest groups and solid research projects with significant results are a priority to improve the indicators of disease burden, health education and a good cost-intervention balance in the near future.

## 3 Conclusions

Scientific research is an indispensable tool for medical students and health personnel in general, since it favors the development of skills and competencies for decision making in clinical practice based on a critical assessment of the available evidence, in order to respond effectively to the needs of patients. Recognition of biases, fallacies, and the advantages and disadvantages of undergraduate research are aspects to consider during the training and design of strategies in evidence-based medicine education and medical research. Evidence is not an absolute, but it is the best tool we have in our hands today.

## Ethical approval

It is not necessary.

## Sources of funding

None.

## Author contribution

All authors equally contributed to the analysis and writing of the manuscript.

## Research registration Unique Identifying number (UIN)

Name of the registry: Not applicable.

Unique Identifying number or registration ID: Not applicable.

Hyperlink to your specific registration (must be publicly accessible and will be checked): Not applicable.

## Guarantor

Sabrina Rahman. Independent University, Dhaka, Bangladesh.

## Declaration of competing interest

None.
